# Supplementary material for: Information provision to caregivers of children with rare dermatological disorders: an international multimethod qualitative study
Source: BMJ Open. 2023 Jul 7;13(7):e070840. doi: 10.1136/bmjopen-2022-070840 (PMC10335406; doi:10.1136/bmjopen-2022-070840)
Supplement: Supplementary data [file bmjopen-2022-070840supp002.pdf]

Supplementary Information 2: Guiding Interview Schedule

Ulster University | Research and Impact

1. Can you tell me a little about the background of your own caregiving situation, including your child's diagnosis?
2. Do you feel there are/were particular stages in your caregiving journey which prove(d) difficult?
3. In your opinion, what ways does/did ichthyosis impact the whole family?
4. How does/did ichthyosis affect your relationships?
5. Can you describe how you feel ichthyosis has affected you as a person (positives and negatives, if any)?
6. Have you ever felt that it is (was) difficult/ stressful?
7. How do you feel you cope(d)?
8. Can you think of any factors or supports which you feel (felt) might be helping you in your caregiving role?
9. Would you be able to describe how you feel about your role as a caregiver as we speak here today?
10. What hopes do you have for caregivers of children with ichthyosis?
11. What expectations do you hold for caregivers of children with ichthyosis?
12. Can you think of any factors or supports which you feel (felt) could have helped you in your caregiving role, if you had a wish-list?
13. Is there anything else that you feel is important to add?
