# Supplementary material for: Information provision to caregivers of children with rare dermatological disorders: an international multimethod qualitative study
Source: BMJ Open. 2023 Jul 7;13(7):e070840. doi: 10.1136/bmjopen-2022-070840 (PMC10335406; doi:10.1136/bmjopen-2022-070840)
Supplement: Supplementary data [file bmjopen-2022-070840supp003.pdf]

Supplementary File 3: Final analytical coding framework for service provided information

Point on care continuum	Themes	Subthemes	First order Coding
**Screening**	Genetic diagnosis and counselling	Information tension<br>Access to specialist care<br>Prenatal testing<br>Family planning<br>Developing countries versus developed countries	Knowledge is power<br>Lost opportunities<br>Experience within pre-diagnostic stage<br>Societal and Healthcare stigma<br>Diagnostic experience<br>Disease validation<br>Disease acceptance<br>Plan for future<br>Roles and Responsibilities<br>Emotional /behavioural reactions<br>Affirming risk perception<br>Emotional reactions
**Active caregiving**	Service provided information at hospital level<br><br>Service provided information at community level	Importance of reporting need<br>Initiating discussion<br>Personal approach<br>Preference for dermatology expertise<br>Continuity of care<br>Shared decision making<br>Structured follow-up care<br>Developing countries versus developed countries<br><br>Increasing point of contact<br>Continuity of care<br>Shared decision making<br>Structured follow-up care<br>Acceptable expertise trade-offs<br>Developing countries versus developed countries	Key personnel involved at each point on care continuum<br>Health beliefs<br>Rarity of disease<br>Healthcare knowledge/awareness<br>Printed information<br>Symptom management & Treatment<br>Care planning<br>Shift in preference of information source and type<br>Communication pathways<br>Trust and treatment adherence<br>Caregiver identification<br>Psychosocial health<br>Male regret<br>Societal stigma<br>Survivorship<br>Stigma in education

	Online medical support groups	Subtype specific groups<br>Instrumental and emotional focus	Inclusion in education<br>Sense of solidarity<br>Sense of mastery<br>Positive emotions<br>Downward comparison<br>Psychosocial management strategies
**Survivorship**	Structured follow-up practical information support	Counselling<br><br><br>Respite<br><br><br><br><br><br><br><br><br><br>Legal matters<br>Financial matters	Male regret over counselling<br>Prepare for future<br>Positive health consequences<br>Opportunity for self-care<br>Building trust<br>Appropriately trained staff<br>Reduced hypervigilance<br>Return to work<br>Positive health consequences<br>Ongoing psychosocial support<br>Bereavement<br>Child independence<br>Adoption<br>Access to healthcare, education, respite<br>Grants, Entitlements, Waivers
