# Supplementary material for: Information provision to caregivers of children with rare dermatological disorders: an international multimethod qualitative study
Source: BMJ Open. 2023 Jul 7;13(7):e070840. doi: 10.1136/bmjopen-2022-070840 (PMC10335406; doi:10.1136/bmjopen-2022-070840)
Supplement: Supplementary data [file bmjopen-2022-070840supp004.pdf]

Supplementary File 4: Overview of caregivers and care recipients in data collection

Table 1: Overview of caregivers in data collection

Participant number	Caregiver gender	Country of residence	Relationship to child	Ichthyosis subtype	Child gender (age in years)	Data collection method
1	F	UK	Mother	Epidermolytic Ichthyosis	M (6)	FG1
2	F	UK	Mother	Epidermolytic Ichthyosis	M (6)	FG1
3	F	ROI	Mother	Netherton's Syndrome	F (1)	FG1
4	F	ROI	Mother	Ichthyosis en confetti	M (14)	FG1
5	F	UK	Mother	Harlequin	F (3)	FG2
6	F	UK	Mother	Lamellar	M (7)	FG2
7	F	UK	Mother	Epidermolytic Ichthyosis	F (8)	FG2
8	F	UK	Mother *	Epidermolytic Ichthyosis	F (17)	FG2
9	F	USA	Mother	Epidermolytic Ichthyosis	1F (17) & 2M (11,14)	FG3
10	F	USA	Mother	Ichthyosis Vulgaris	M (4)	FG3
11	F	USA	Mother	Epidermolytic Ichthyosis	F (2)	FG3
12	F	USA	Mother	Epidermolytic Ichthyosis	M (5)	FG3
13	F	USA	Mother	Netherton's syndrome	F (14)	FG3
14	F	USA	Mother	Epidermolytic Ichthyosis	M (3)	FG3
15	M	USA	Father	Harlequin	F (25)	FG4
16	M	USA	Father	Harlequin & Lamellar	2M (5,13)	FG4
17	M	USA	Father	Epidermolytic Ichthyosis	M (18)	FG4
18	F	ROI	Mother	Epidermolytic Ichthyosis	M (13)	FG5
19	F	NI	Mother	X-Linked	2M (6, 14)	FG5
20	F	USA	Mother	Epidermolytic Ichthyosis	M (4)	FG5
21	F	USA	Mother	X-Linked	M (11)	FG5
22	F	USA	Mother	Lamellar	M (4)	FG5
23	F	Australia	Mother	Congenital ichthyosiform erythroderma (CIE)	F (<1)	FG6
24	F	Australia	Mother	Harlequin	F (<1)	FG6
25	F	Philippines	Mother	Epidermolytic Ichthyosis	F (1)	FG6
26	F	ROI	Mother	Lamellar	M (7)	FG6
27	F	ROI	Mother	Lamellar	M (2)	FG6
28	F	Canada	Mother	X-Linked	M (7)	Interview
29	F	Switzerland	Mother	Epidermolytic Ichthyosis	F (1)	Interview

30	M	USA	Father	Epidermolytic Ichthyosis	2F (11, 13)	Interview
31	M	ROI	Father	Harlequin	F (7)	Interview
32	F	India	Mother	Lamellar	F (19)	Interview
33	F	ROI	Mother	Epidermolytic Ichthyosis	F (3)	Interview
34	M	USA	Father	Harlequin	M (1)	Interview
35	F	USA	Grandmother	Harlequin	M (6)	Email
36	M	Netherlands	Father*	X-Linked	F (2)	Email
37	F	USA	Mother	X-Linked	2M (1, 4)	Email
38	F	Greece	Mother	Congenital ichthyosiform erythroderma (CIE)	1F (3) & 1M (6)	Email
39	M	ROI	Father	Harlequin	F (<1)	Email
**Legend:** UK United Kingdom, ROI Republic of Ireland, USA United States of America, NI Northern Ireland, M Male, F Female, FG Focus Group *Patient Caregiver						

Table 2: Socio-demographic status of caregivers in qualitative study

	**Female n (%)**	**Male n (%)**
**Total number of participants (n=39)**	31 (79)	8(21)
**Age range in years**		
18-24	1 (2.5)	
25-34	9 (24)	2 (5)
35-44	15 (38)	2 (5)
45-54	4 (10)	3 (8)
55-64	1 (2.5)	
65-74	1 (2.5)	1(2.5)

Table 3: Care Recipient Characteristics

**Age range in years of affected children**	**Female children (n)**	**Male children (n)**
<1	3	0
1-4	8	8
5-12	3	12
13-19	5	6
20+	1	0
**Total**	**20**	**26**
