# Supplementary material for: Information provision to caregivers of children with rare dermatological disorders: an international multimethod qualitative study
Source: BMJ Open. 2023 Jul 7;13(7):e070840. doi: 10.1136/bmjopen-2022-070840 (PMC10335406; doi:10.1136/bmjopen-2022-070840)

Supplementary File 5: Overview of themes and subthemes identified for service provided information support at points on the care continuum

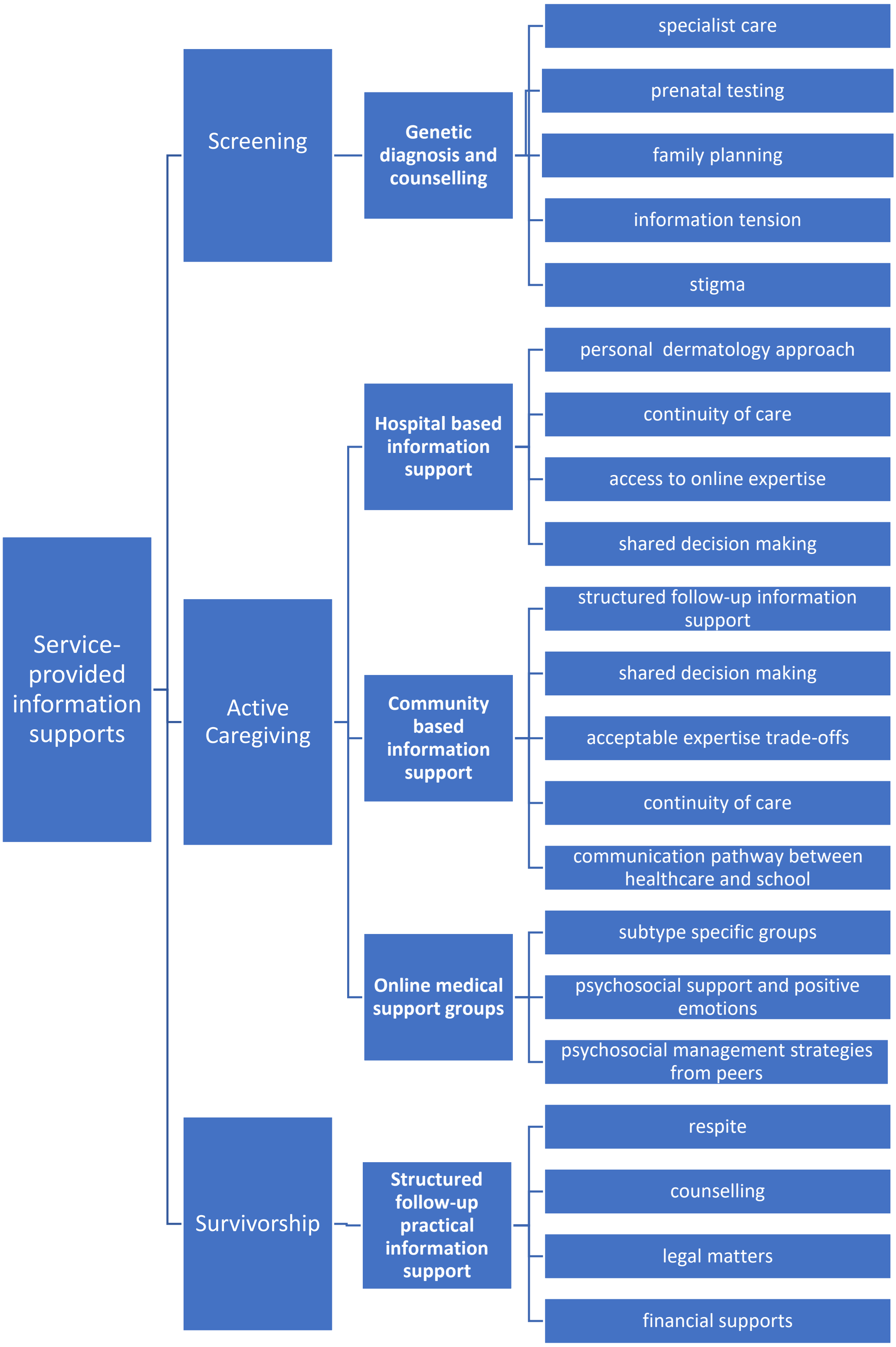

Supplement: Supplementary data [file bmjopen-2022-070840supp005.pdf]
