# Supplementary material for: Information provision to caregivers of children with rare dermatological disorders: an international multimethod qualitative study
Source: BMJ Open. 2023 Jul 7;13(7):e070840. doi: 10.1136/bmjopen-2022-070840 (PMC10335406; doi:10.1136/bmjopen-2022-070840)
Supplement: Supplementary data [file bmjopen-2022-070840supp006.pdf]

Supplementary File 6: Recommendations for optimising service-provided information support in dermatology

National / State level

(1) Increased lobbying by dermatology teams at both national and international level for improved recruitment, dermatological / psychology training and resourcing.
(2) Fund a working party to review current practice and help finds solutions to any perceived barriers to care, with the aim of establishing a National clinical service.
(3) The development of a consortium of experts across Europe to help provide clinical guidelines.
(4) The development of a national Mentorship Scheme whereby the 'expert' hospitals are reimbursed for offering education and support to other target hospitals to improve access to care, and develop local protocols and standard operating procedures. This could be achieved via conferences, hospital site visits, and teleconferences. It would be additionally beneficial for a member of the mentorship team to provide on-site support during treatment delivery for the first patients affected with the more severe diseases and/or subtypes.
(5) The development of best practice clinical guidelines for the management of the skin disease using an international evidence-based collaborative approach which incorporates research-based evidence, professional expertise, charity organisations and patient/caregiver values.
(6) Distribution of a disease-specific e-factsheet, created by clinical specialists, to all Neonatal Intensive Care Units (NICU) and paediatric units
(7) Online exchange of dermatological expertise within and between different healthcare systems, using telecommunications including artificial intelligence (Smartphone App, ICD coding). This is particularly crucial for clinicians in developing countries and/or at the birth of an affected child.
(8) Improved continued professional development for healthcare staff to increase awareness on:
    a. improving caregiver identification and reducing potential barriers
    b. promoting awareness of the physical and psychosocial impact of living with and caregiving for a child with skin disease
    c. importance of using positive language when describing both the affected child and/or disease
    d. reducing unintentional healthcare stigma of visual and/or genetic conditions
    e. available information supports for affected child(ren) and family
    f. over-reliance of medication to treat caregiver emotional health

Healthcare level

(1) Service-provided information support should be relevant, personalised to the child's disease and/or subtype, explained comprehensively and communicated well by the same physician on each occasion where possible.
(2) To provide the caregiver with extensive printed information with respect to their child's diagnosis, symptom management, available treatment and prognosis to encourage shared decision-making power. Focus should be given to supporting older caregivers and/or those with poor physical health with such information.

(3) Appointment of a care co-ordinator to be the first point of contact for the family in all aspects of social and healthcare
(4) Shared decision making could be encouraged through the
    a. development of a care plan for the affected child prior to initial hospital discharge, which would be subsequently reviewed in joint-partnership and at regular intervals to reflect changing caregiver information needs
    b. development of an information 'passport' which could accompany the affected child at key life transitions, including acute hospital admissions, airports and/or change of class
(5) First treatment evaluation and all structured follow-up visits should be performed by dermatology specialists to develop trust and promote treatment adherence, particularly with younger caregivers. Where this is not possible:
    a. tele-dermatology or periodic follow-up visits should be made available with the same physician every time to increase certainty and validation of disease symptoms
    b. quick and easy clinical reviews should be accommodated when issues of concern arise between visits, either face-to-face or online
    c. a liaison nurse should be appointed to the family to help ensure structured follow-up care and promote open and transparent communication pathways between hospital and community healthcare teams.
(6) A multi-disciplinary information approach would help facilitate and promote the timely exchange of practical information support, particularly on legal matters, respite provision, counselling and entitlements.
(7) Provision of structured follow-up care to promote successful inclusion in childcare and/or education:
    a. timely visit from relevant dermatology clinician(s) and/or early intervention team to relevant setting to improve awareness of the condition at *whole-school* level, allow for modifications/accommodations, share information resources with all staff and support relevant personnel in applying for necessary resources to ensure mainstream inclusion, such as teaching assistant support.
(8) Provision of an integrated eHealth needs assessment tool which caregivers can regularly complete in their own time and which would allow healthcare professionals to update/identify, triage and support caregivers' needs and preferences in a timely manner. Essential to assess caregivers' information support needs in the areas of medical training, symptom management, treatment and products, financial matters,
(9) Provision of medical and psychosocial information support for families of palliative patients with skin disease, particularly in relation to nature, course, outcome and available supports
(10) Automatic and timely provision of psychosocial support for the affected child, caregiver and family unit.

Online Support Groups

(1) Improved information exchange within online support groups through the:
    a. regular updating of available medical information
    b. development and monitoring of subtype specific subgroups

c. advertisement of research opportunities, clinical trials and recent relevant research publications
d. development and assessment of instrumental and psychosocial interventions
e. facilitation of subtype specific caregiver group social discussion calls
f. establishment of a working party with healthcare providers to work though academic publications, to translate information into feasible, tangible clinical workflows and care plans
